# *Stenotrophomonas maltophilia* endogenous endophthalmitis in an immunocompetent eye in Australia

**DOI:** 10.1186/s12886-025-03990-4

**Published:** 2025-03-28

**Authors:** Cadric Gunaratnam, Ario A. Wilson-Pogmore, Travers Weaver

**Affiliations:** 1 Department of Ophthalmology, Division of Surgery, Princess Alexandra Hospital, 199 Ipswich Rd, Woolloongabba, Brisbane, QLD 4215 Australia; 2 Faculty of Medicine, University of Queensland, Brisbane, Australia

**Keywords:** Endogenous endophthalmitis, Stenotrophomonas maltophilia, Xanthomonas maltophilia

## Abstract

**Background:**

Background *Stenotrophomonas maltophilia* has increasingly become a significant hospital-acquired pathogen due to its multi-resistance and ability to colonize surfaces. While ocular infections caused by *S. maltophilia* are documented, endogenous endophthalmitis (EE) is rare with only six cases reported in adults, mostly in immunocompromised individuals from developing countries. Here, we present the first documented case of *S. maltophilia*-associated EE in an immunocompetent adult in Australia. A 43-year-old female presented with a two-day history of painful vision loss in her left eye. There was no preceding trauma, systemic illness, or history of intravenous drug use. Notably, she worked as a marine biology researcher and had recently been visiting the hospital often to see family. Examination revealed severe anterior chamber inflammation, hypopyon, and vitritis. Initial treatment included hourly dexamethasone 0.1% eye drops while awake, atropine 1% three times daily, a single vitreous tap, and injection of intravitreal vancomycin (1 mg/0.1mL), ceftazidime (2.25 mg/0.1mL), and voriconazole (0.05 mg/0.1mL). Cultures confirmed *Stenotrophomonas maltophilia*, and oral trimethoprim-sulfamethoxazole (160 mg/800 mg) 1.5 tabs three times daily was initiated but later switched to moxifloxacin 400 mg daily due to neutropenia. One-month post-discharge, the patient developed an inferior retinal detachment requiring vitrectomy with silicone oil. At six months, her visual acuity improved to 6/18.

**Conclusion:**

*S. maltophilia*-associated EE is a rare clinical condition, especially in the absence of systemic symptoms. Despite the poor prognosis typically associated with endophthalmitis, this case resulted in a relatively good visual outcome (BCVA 6/18). This report highlights the necessity for further research to elucidate the epidemiology, risk factors, and optimal management strategies for this rare ocular condition.

## Background

*Stenotrophomonas maltophilia*, previously known as *Pseudomonas maltophilia* and *Xanthomonas maltophilia*, is a gram-negative rod found in soil and water. It has become a notable hospital-acquired pathogen due to multi-resistance and ability to colonize surfaces, including plastic, glass, and polytetrafluoroethylene [1, 2]. The prevalence of hospital-acquired infections attributed to *S. maltophilia* is increasing, especially among immunocompromised patients, and it is associated with significant fatality rates [1]. *S. maltophilia* can cause a wide range of ocular infections including conjunctivitis, keratitis, scleritis, dacryocystitis, pre-septal cellulitis, and endophthalmitis [2, 3]. At the time of writing, most cases of *S. maltophilia* endophthalmitis described in the literature are associated with surgery or trauma, with endogenous endophthalmitis (EE) being rare [4]. To date there have been six documented cases of *S. maltophilia-*associated EE in adults [2–5]. Among these cases, three occurred in otherwise healthy individuals [2–5]. Notably, all previously documented cases have been isolated in India. Here we present, to the our knowledge, the first case of *S. maltophilia-*associated EE documented in a healthy adult individual in Australia.

## Case presentation

A 43-year-old Caucasian female presented to the Princess Alexandra Hospital (Brisbane) Eye Casualty with a painful deterioration of vision in her left eye over two days. There was no preceding fever or additional complaints. Her past ocular history was unremarkable, with no history of trauma or intravenous drug use. Notably, she worked as a marine biology researcher and had recently been visiting the hospital often to see family.

**Fig. 1:**
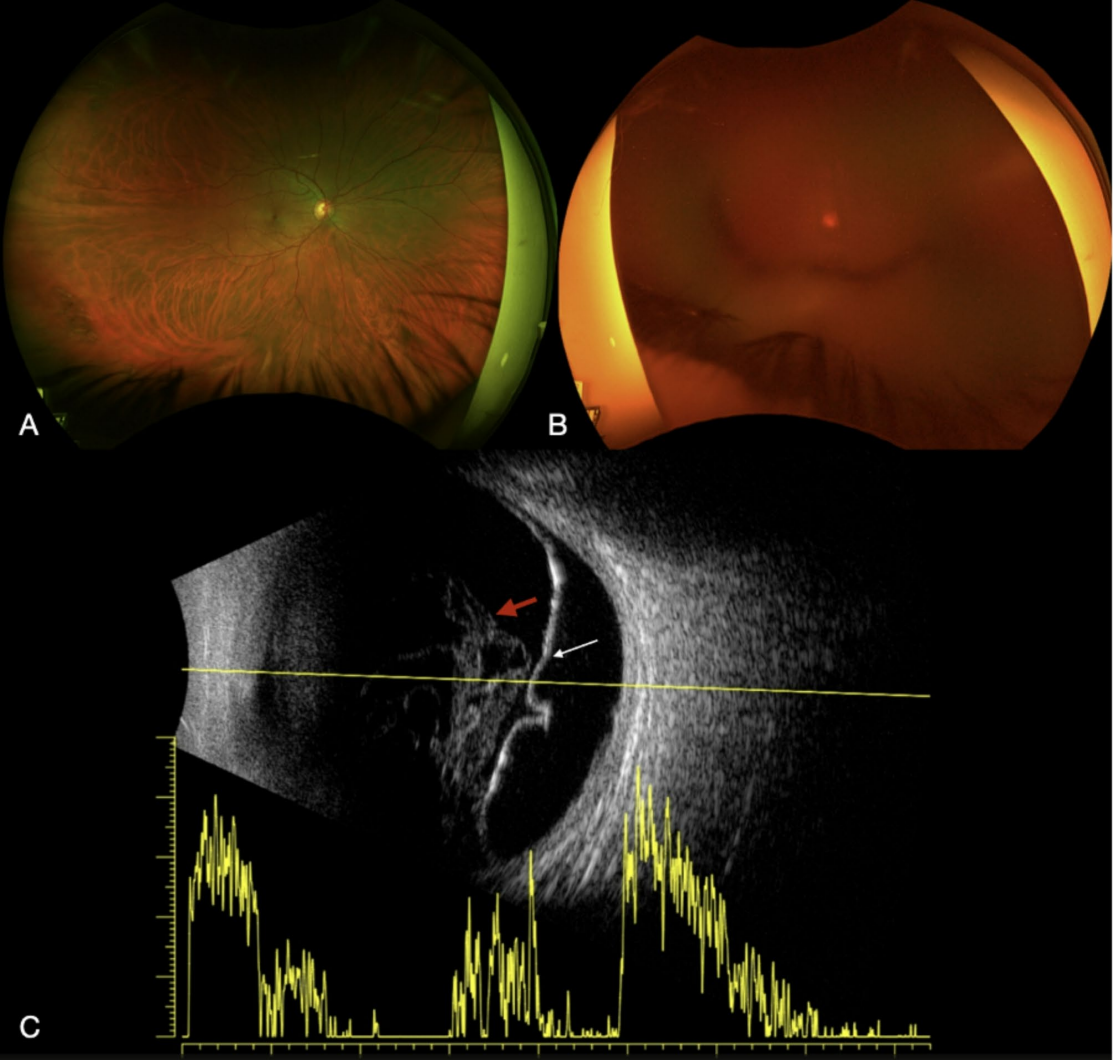
Colour fundus photo illustrating normal posterior pole of the right eye (**A**) and dense vitritis in the left eye (**B**). B Scan ultrasonography illustrating membranous hyperechoic changes within the vitreous (red arrow), along with thickened posterior hyaloid (white arrow)(**C**)

Upon presentation, her unaided right eye visual acuity (VA) was 6/6, while the left VA was hand movements (HM) with no improvement using a pinhole (PH). Intraocular pressures (IOP) were 15 mmHg in the right eye and 21 mmHg in the left. Her left eye conjunctiva had grade 3 injection with no chemosis, there was mild corneal oedema, and diffuse fine keratic precipitates. There was a severe inflammatory response in the anterior chamber (AC) with 4 + cells and near-complete posterior synechiae. She was phakic with fibrinous deposits on the lens. The posterior structures and fundus could not be visualised. B-scan ultrasonography was unremarkable showing no evidence of vitritis. Her right eye examination was unremarkable (Fig. 1A).

A provisional diagnosis of severe left acute anterior uveitis was made. The patient was started on hourly dexamethasone 0.1% eye drops while awake and atropine 1% three times daily. A uveitic panel was ordered including CRP, ESR, HLA-B27, ACE, ANA, Syphilis serology, Quantiferon Gold, and chest x-ray.

On review, 48 h following her initial presentation, the patient had reported symptomatic improvement, however her unaided VA in the left eye remained HM. Further examination of her left eye revealed a new 0.8 mm hypopyon, persistent severe AC inflammation, and the fundus was still not visible (Fig. 1B). Repeat B-scan ultrasonography revealed new membranous hyperechoic changes within the vitreous and thickened posterior hyaloid concerning for vitritis (Fig. 1C).

A provisional diagnosis of left endogenous endophthalmitis was made and the patient was admitted. A vitreous sample was taken for microscopy, cultures, and fungal studies. The patient received an intravitreal injection of vancomycin (1 mg/0.1mL), ceftazidime (2.25 mg/0.1mL), and voriconazole (0.05 mg/0.1mL). Topical dexamethasone and atropine were continued, while chloramphenicol 0.5% was added. The patient was commenced on intravenous (IV) vancomycin (25 mg/kg) and ceftazidime 2 g three times daily. Initial gram stain demonstrated gram-negative bacilli.

The uveitic screen, urine, and blood cultures were unremarkable. Two days after admission, vitreous cultures identified *Stenotrophomonas maltophilia* sensitive to co-trimoxazole, moxifloxacin, and ceftazidime. In collaboration with Infectious Diseases (ID), the intravenous antibiotics were discontinued, and the patient was commenced on oral trimethoprim-sulfamethoxazole (160 mg/800 mg) 1.5 tabs three times daily (12 mg/kg/day) for a total course of four weeks. An additional intravitreal injection of ceftazidime (2.25 mg/0.1mL) was also administered following poor clinical progression. The patient was discharged five days following admission. Her left VA had improved to Count Fingers (CF) with an IOP of 8 mmHg.

Following two weeks of trimethoprim-sulfamethoxazole, the patient became neutropenic and developed bilateral cervical lymphadenopathy. Treatment was promptly changed to moxifloxacin 400 mg daily. Four weeks post-discharge, her left eye VA improved to 6/36. Her conjunctival injection had resolved, and AC inflammation significantly improved with only trace cells remaining. Her fundal view improved, showing a clear view of the superior retina, while central vitreous condensations and large inferior vitreous opacities could be appreciated. Unfortunately, the patient had developed an inferior retinal detachment through the clinical course necessitating urgent pars plana vitrectomy with scleral buckle and silicone oil.

Six months post-retinal detachment repair, the patient’s endophthalmitis had resolved with no residual inflammatory signs. Noting a visually significant post-vitrectomy cataract, her unaided vision was 6/24 improving with PH to 6/18.

## Discussion

EE typically presents with systemic features such as fever or malaise. Often, patients have associated conditions such as diabetes mellitus, urinary tract infections, immunosuppression, or recent hospitalizations [6]. However, a recent review of EE suggested that up to 18% of patients with endogenous bacterial endophthalmitis did not exhibit significant constitutional symptoms [3]. 

While post-surgical and post-traumatic endophthalmitis caused by *S. maltophilia* is well documented, EE remains rarer. To date, only seven cases of *S. maltophilia*-associated EE have been reported. All of which have occurred in patients from low-socioeconomic areas. Notably, two reported cases have occurred in otherwise healthy individuals in the absence of ocular trauma, surgery, or systemic involvement [2–5]. In both instances, blood and urine cultures were unremarkable, and no source of infection was identified, similar to our patient’s presentation. High prevalence of protein energy malnutrition-related immunocompromise has been hypothesised to be contributing to EE by a microorganism with low virulence [7]. 

Our patient is fit and well with no nutritional deficiency or immunocompromising conditions. We hypothesize that the patient may have been exposed to an *S. maltophilia* reservoir from frequent hospital visits at the time of presentation or exposure in the aquaculture industry.

Treatment guidelines for *S. maltophilia* infections are limited due to scarce clinical data [8]. Historically, trimethoprim/sulfamethoxazole have been considered the standard treatment with fluoroquinolones, tetracyclines, and selected β-lactams commonly used as alternatives [8]. Variable antimicrobial susceptibility have been described with a majority of strains being sensitive to second and fourth-generation fluoroquinolones, chloramphenicol, aminoglycosides, and cephalosporins (Table 1).

**Table 1:** Clinical and microbial characteristics of documented patients with Stenotrophomonas maltophilia endogenous endophthalmitis in current literature. [2–5]

Reference	#	Age	Sex	Eye	Initial BCVA	Sensitivity	Resistance	Final BCVA
Chhablani et al.	1	35	M	RE	LP	ami, cef, cpl, cip, gen, gat, mox, ofl, tic, tob, van	pip	LP
Chhablani et al.	2	22	F	LE	HM	ami, gen, gat, mox, cip, cpl, van, ofl, pip, tob	tic	6/9
Chhablani et al.	3	35	M	RE	6/95	ami, cip, cpl, cef, gen, mox, ofl, pip, tob, tic, van		6/6
Chhablani et al.	4	30	M	LE	LP	gat, mox, cip, cpl, ofl, pip	ami, cef, gen, tob, van	6/24
Suhan et al.	5	26	F	LE	LP	ami, cip, gen	cef, cpl	6/9
Bilgic et al.	6	42	M	RE	LP			6/24
Gunaratnam et al.	7	43	F	LE	HM	sxt, cef, mox		6/18

## Conclusion

*S. maltophilia*-associated EE is an exceptionally rare clinical entity. This case report contributes to the sparse body of literature documenting this atypical presentation, particularly in the absence of systemic symptoms or identifiable risk factors. The patient’s favorable visual outcome, achieving a BCVA of 6/18 is noteworthy, considering the generally poor visual prognosis associated with EE. While standardized management guidelines for *S. maltophilia* associated endophthalmitis remain undefined, our experience suggests that fluoroquinolones or aminoglycosides may be effective initial therapeutic options. Further research is necessary to elucidate the epidemiology, risk factors, and optimal treatment strategies for this uncommon form of endophthalmitis.

## Data Availability

This manuscript does not report data generation or analysis.

## Abbreviations

EE: Endogenous endophthalmitis
PH: Pinhole
IOP: Intraocular pressures
AC: Anterior chamber
IV: Intra-venous
CF: Count Fingers
VA: Visual acuity
RE: Right eye
LP: Light perception
van: Vancomycin
cef: Ceftazidime
ami: Amikacin
cpl: Chloramphenicol
gen: Gentamicin
gat: Gatifloxacin
mox: Moxifloxacin
ofl: Ofloxacin
tic: Ticarcillin
tob: Tobramycin
pip: Piperacillin
LE: Left eye
HM: Hand motions
cip: Ciprofloxacin
sxt: Co-trimoxazole
